# Supplementary material for: Oxidative Stress-Mediated DNA Damage Induced by Ionizing Radiation in Modern Computed Tomography: Evidence for Antioxidant-Based Radioprotective Strategies
Source: Antioxidants (Basel). 2025 Sep 4;14(9):1085. doi: 10.3390/antiox14091085 (PMC12466383; doi:10.3390/antiox14091085)
Supplement: Supplementary file 1 [file antioxidants-14-01085-s001.zip › antioxidants-3798217-supplementary.pdf]

antioxidants

MDPI

Article

Supplementary Material: Oxidative Stress-Mediated DNA Damage Induced by Ionizing Radiation in Modern Computed Tomography: Evidence for Antioxidant-Based Radioprotective Strategies

Table S1. CT scanning parameters.

Parameters	Non-Contrast Phase	Contrast-Enhanced Phase
kV (reference)	100	100
mAs (reference)	180	180
Dose optimization level	3	7
Rotation time (s)	0.5	0.5
Delay (s)	2	80
Pitch	1	1
Collimator (mm)	128 × 0.6	128 × 0.6

Table S2. Characteristics of the CT exposure (TEXP).

Values are presented as mean ± SD, median (interquartile range). CE, contrast-enhanced; CT, computed tomography; CTDIvol, CT dose index; DLP, dose-length product.

**Characteristics**	**BEXPOSURE**	
	n	**=18**
Vintage between the non CE phase CT acquisition and blood sample #2	11	(3, 16)
Vintage between the CE phase CT acquisition and blood sample #2	8	(1, 9)
CT acquisition parameters and radiation dose		
kV, non-CE phase	100	± 0
kV, CE phase	100	± 0
mAs, non-CE phase	131	(112, 159)
mAs, CE phase	131	(116, 181)
Rotation time, non-CE phase	0.5	± 0
Rotation time, CE phase	0.5	± 0
CTDIvol, mGy	1020	(905, 1342)
DLP, mGy*cm	381	(347, 571)
Patient-specific effective dose, mSv	6.7	(6.2, 9.0)

Values are presented as mean ± SD, median (interquartile range). CE, contrast-enhanced; CT, computed tomography; CTDIvol, CT dose index; DLP, dose-length product.

Table S3. Characteristics of the CT exposure (T_{EXP}) by patient.

**Patient Number (anonym)**	**$B_{EXPOSURE}$ group Effective Dose in mGy**
1	6.68
2	11.27
3	6.29
4	9.14
5	6.77
6	5.73
7	7.68
8	7.39
9	6.61
10	6.33
11	8.98
12	6.67
13	11.81
14	4.38
15	6.86
16	9.98
17	5.19
18	6.02
